# Feasibility of cognitive rehabilitation in patients with advanced multiple sclerosis: A pilot study

**DOI:** 10.1177/20552173211064473

**Published:** 2021-12-10

**Authors:** Stefanos E Prouskas, Nancy D Chiaravalloti, Neeltje Kant, Karlene K Ball, Vincent de Groot, Bernard MJ Uitdehaag, Jeroen JG Geurts, Elizabeth A Kooij, Hanneke E Hulst

**Affiliations:** Amsterdam UMC, Vrije Universiteit Amsterdam, Department of Anatomy & Neurosciences, MS Center Amsterdam, Amsterdam Neuroscience, The Netherlands; Nieuw Unicum, The Netherlands; Neuropsychology and Neuroscience Laboratory, Kessler Foundation, USA; Department of Physical Medicine and Rehabilitation, Rutgers New Jersey Medical School, USA; Nieuw Unicum, The Netherlands; Department of Psychology, 9968 University of Alabama at Birmingham, USA; Amsterdam UMC, Vrije Universiteit Amsterdam, Department of Rehabilitation Medicine, MS Center Amsterdam, Amsterdam Movement Sciences, Amsterdam, The Netherlands; Amsterdam UMC, Vrije Universiteit Amsterdam, Department of Neurology, MS Center Amsterdam, Amsterdam Neuroscience, Amsterdam, The Netherlands; Amsterdam UMC, Vrije Universiteit Amsterdam, Department of Anatomy & Neurosciences, MS Center Amsterdam, Amsterdam Neuroscience, The Netherlands; Nieuw Unicum, The Netherlands; Amsterdam UMC, Vrije Universiteit Amsterdam, Department of Anatomy & Neurosciences, MS Center Amsterdam, Amsterdam Neuroscience, The Netherlands

**Keywords:** Cognition, multiple sclerosis, progressive, rehabilitation, feasibility

## Abstract

**Background:**

The feasibility of cognitive rehabilitation is rarely investigated in patients with advanced multiple sclerosis.

**Methods:**

Eighteen patients with advanced multiple sclerosis (median EDSS = 7.5) were randomized into restorative or compensatory cognitive rehabilitation. Feasibility was determined by adherence rate, completion rate, patient satisfaction, self-reported fatigue, training difficulty, and training duration.

**Results:**

Adherence rates and completion rates were over 70%, and patients were highly satisfied in both groups. Energy levels decreased minimally during the sessions (pre = 6.9 vs post = 6.4). Training difficulty (4.6) and duration (5.7) were close to ideal (scale 1–10, 5 = ideal).

**Conclusions:**

Cognitive rehabilitation, with minor adjustments, appears feasible in patients with advanced multiple sclerosis.

## Introduction

Cognitive impairment is present in 43–65% of patients with multiple sclerosis (MS),^1^ and becomes more frequent and more severe in patients with advanced MS (secondary and primary progressive MS).^2^

Cognitive rehabilitation generally consists of either restorative training (i.e. retraining cognitive functions) or compensatory training (i.e. strategies to compensate for daily life restrictions that result from cognitive impairment). Meta-analyses show that computerized cognitive rehabilitation in particular seems promising, with moderate overall effect sizes (Hedge's *g* *=* 0.30).^3^ Yet, the vast majority of such cognitive rehabilitation studies focus on relatively low-disability MS (EDSS = 3.5),^3,4^ while compensatory training remains the most frequently used option for advanced MS in clinical practice.

Whether cognitive rehabilitation programs are suitable in patients with advanced MS remains unknown.^4^ It can be argued that the increased disease burden (e.g. fatigue, motor problems) may hamper the applicability of cognitive rehabilitation.^5^ As such, before setting up large studies examining the effectiveness of cognitive rehabilitation in advanced MS, the first step is to determine its feasibility. Therefore, we investigated the ability of patients with advanced MS to participate in two forms of cognitive rehabilitation (compensatory and restorative training).

## Methods

### Participants

Eighteen patients with advanced MS (age *mean* *=* 58.1  ±  5.0 years, EDSS median = 7.5, disease duration *mean* *=* 20.6  ±  6.2 years, 13 SPMS, 5 PPMS) were recruited from Nieuw Unicum, a center which houses over 140 patients who due to their MS-related disabilities require intensive care and 24/7 monitoring. Advanced MS was defined as an EDSS score higher than 6.0, in accordance to previous studies.^6^ Patients were randomized to either a compensatory memory strategies (CST) group intervention (*n* = 9) or a speed of processing training (SPT) individualized intervention (*n* = 9). Both interventions were given at the patient living facility.

The study was approved by the Medical Ethical Committee of the Amsterdam University Medical Centers. Written informed consent was obtained prior to participation.

### Interventions

#### Compensatory memory strategies training

CST is a group intervention that is provided *standard of care* in Nieuw Unicum. The program consists of compensation strategies to help patients cope with cognitive impairment in daily life (Supplemental Table S1).^7^ According to protocol,^7^ sessions were held once a week for 90 min over a total period of 9 weeks.

#### Speed of processing training

SPT is an individual adaptive computerized cognitive rehabilitation program that focuses on information processing speed and proven effective in patients with relapsing remitting multiple sclerosis (RRMP).^8^ According to protocol,^8^ patients trained twice a week, 60 min per session, for 5 weeks. To avoid interference of motor symptoms, the researcher (S.P.) controlled the mouse and registered the answers that patients orally communicated.

### Feasibility

Feasibility was determined by recruitment rate, dropout rate, adherence rate (percentage of sessions attended), completion rate (percentage of time spent training per session), and scores on the Client Satisfaction Questionnaire (CSQ-8). Based on these variables, it is possible to make a comparison between the feasibility of CST (compensatory training) and SPT (restorative training).

Feasibility of the SPT was additionally assessed using a self-report and observant (S.P.) questionnaire, which rated the following items on a scale of 1–10: motivation, fatigue, concentration, instructions clarity, accessibility of materials, training difficulty, and training duration. The questionnaires were filled out for each session and for the training as a whole.

### Statistical analysis

Descriptive statistics were obtained using SPSS (IBM Corp. Released 2013. IBM SPSS Statistics for Windows, Version 22.0. Armonk, NY: IBM Corp.).

## Results

### Patients

**Figure 1.**
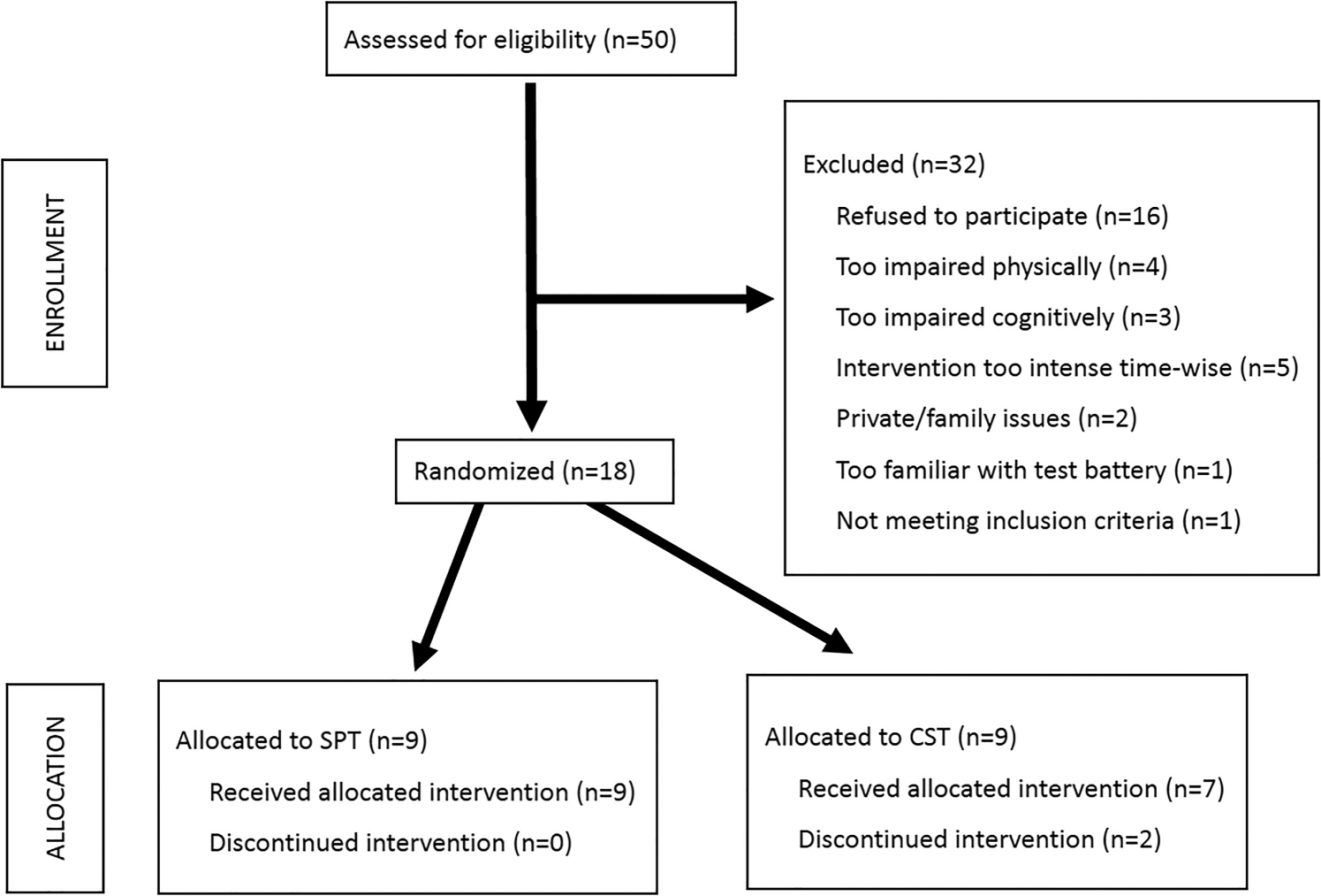
Flowchart of participation.

Fifty patients were contacted for participation, out of which 18 agreed to participate, resulting in a 36% recruitment rate (Figure 1). Table 1 presents the demographic data and feasibility outcomes.

**Table 1.** Baseline characteristics of the patients per group. Nonparametric statistical analyses revealed no statistically significant group differences.

	All (*n* = 18)	SPT (*n* = 9)	CST (*n* = 9)	
	Mean (*SD*)	Mean (*SD*)	Mean (*SD*)	* p*
Age [years (*SD*)]	58.1 (5.0)	56.9 (5.5)	59.3 (4.4)	0.506
Sex				
Male	7 (38.9%)	3 (33.3%)	4 (44.4%)	0.629
Female	11 (61.1%)	6 (66.7%)	5 (55.6%)	
Education [median (IQR)]	6^1^	5^2^	6^1^	
Min	4	4	4	0.599
Max	7	7	6	
MS type				
SPMS	13 (72.2%)	6 (66.7%)	7 (77.8%)	0.065
PPMS	5 (27.8%)	3 (33.3%)	2 (22.2%)	
Disease duration [years (*SD*)]	20.6 (6.2)	19.7 (5.7)	21.4 (7.0)	
Min	10	13	10	0.535
Max	31	31	31	
EDSS [median (IQR)]	7.5 (0.0)	7.5 (0.3)	7.5 (0.3)	
Min	6.5	7.0	6.5	0.43
Max	8.5	8.5	8.5	

EDSS: expanded disability status scale; SPMS: secondary progressive multiple sclerosis; PPMS: primary progressive multiple sclerosis.

### CST

In the CST group (*n* = 9), two patients dropped out within the first two weeks. The remaining seven patients finished the program with 96.8% average adherence rate and 96.3% average completion rate. Patients were mildly satisfied with the CST (CFQ-8 score 24.0  ±  2.9).

### SPT

There were no dropouts in the SPT group. This group had 84.4% average adherence rate and 68.2% average completion rate. Four patients indicated having difficulty maintaining concentration in the last 15 min of some sessions. The heatwave (temperatures of >30°C) negatively influenced the adherence and completion rates (the CST took place in an air-conditioned room). Patients indicated that they were overall satisfied with the intervention: mean CSQ-8 score was 28.1  ±  1.9. Table 2 presents the results of the feasibility questionnaires. Patients’ overall fatigue levels were moderate (*mean* *=* 4.5  ±  2.2 (optimal score  =  5)), and the training sessions minimally decreased patients’ energy level (pre *mean* *=* 6.9  ±  1.0, post *mean* *=* 6.4  ±  0.9). Patients reported a more than satisfactory level of motivation per session (*mean* *=* 7.7  ±  1.3) and hold their concentration relatively well throughout the training program (*mean* *=* 7.0  ±  1.7).

**Table 2.** Results of feasibility questionnaires.

SPT evaluation after session 1	Self-report (*n* = 9)	Researcher observation (*n* = 9)	SPT evaluation after completion of entire training	Self-report (*n* = 8)	Researcher observation (*n* = 9)
General motivation	7.9 (1.3)^b^	6.8 (2.2)^b^	Training fatigue	4.5 (2.2)^a^	6.1 (2.3)^a^
Training level		5.3 (2.4)^c^	Training concentration	7.0 (1.7)^b^	6.2 (2.5)^b^
Patient burden		4.1 (2.8)^a^	Instructions clarity	9.5 (0.8)^b^	9.4 (0.9)^b^
			Training difficulty	4.6 (2.3)^c^	3.9 (2.5)^c^
			Accessibility of materials	9.1 (1.2)^b^	10.0 (0.0)^b^
			Training length evaluation	5.7 (1.1)^c^	6.0 (1.2)^c^
**Self-reports at each session**	**M (SD) (*n* = 9)**		**Researcher observations throughout each session**		**M (SD) (*n* = 9)**
Energy level pre-session	6.9 (1.0)		Observed fatigue level		4.3 (1.6)
Energy level post-session	6.4 (0.9)		Observed motivation level		7.5 (1.4)
Motivation level pre-session	7.7 (1.3)		Observed concentration level		7.4 (1.0)
Session performance evaluation	7.2 (0.6)				

^a^ Lower scores are better.

^b^ Higher scores are better.

^c^ A score of 5 indicating ideal; 0 is too difficult/short, 10 is too easy/long

## Discussion

The aim of this study was to determine the *feasibility* of cognitive rehabilitation in patients with advanced MS, a group that is often excluded from research despite extensive cognitive decline.^4^ The current results may encourage and guide future research on cognitive rehabilitation in advanced MS that is urgently warranted (https://www.progressivemsalliance.org/what-we-do/speed-clinical-trials).

**Figure 2.**
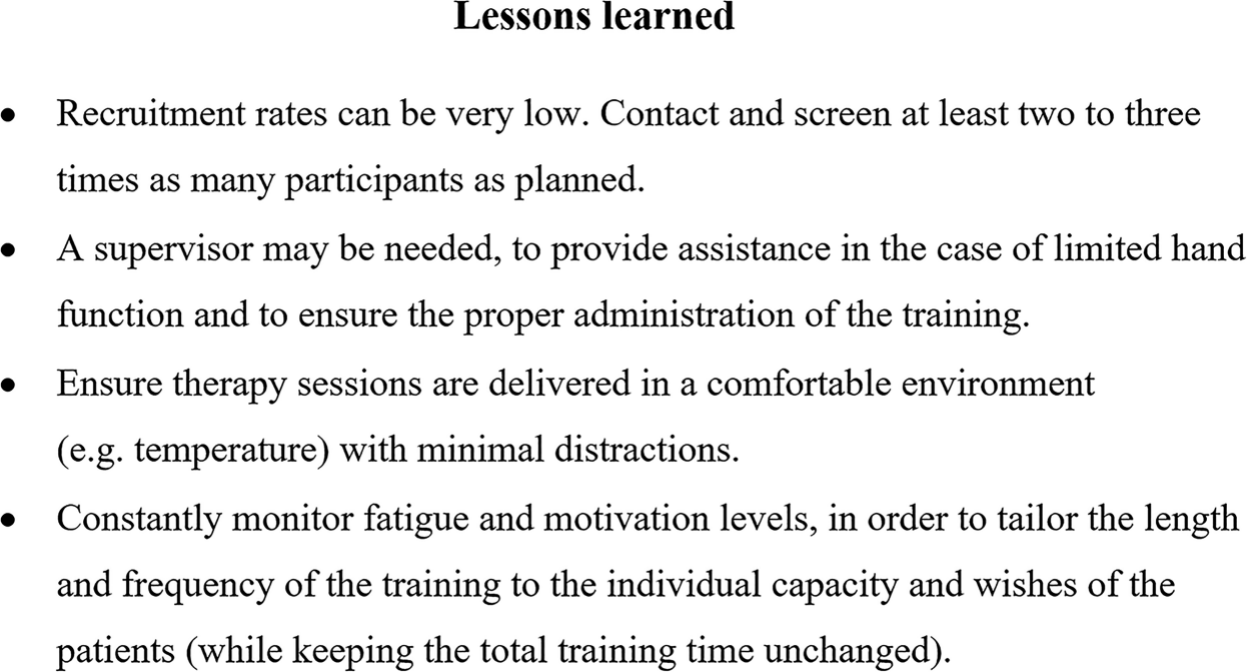
Lessons learned regarding cognitive rehabilitation in advanced MS.

It should be noted that the current findings are based on a small number of rare patients hampering generalization to the general MS population. However, for cognitive rehabilitation in this patient group, we have learned the following (Figure 2): when setting up a cognitive rehabilitation study in advanced MS, low recruitment rates (36%) should be anticipated, requiring three times more patients to be initially contacted to obtain sufficient sample sizes. The SPT showed lower adherence (84.4% versus 96.8%) and completion rates (68.2% versus 96.3%) compared to CST, which might be explained by the intervention type (group versus one-on-one) and the frequency of sessions (twice a week versus once every two weeks).^9^ The lower completion rates found in the current study might additionally be a consequence of external factors (i.e. heatwave). Note that these adherence rates are only valid for at-home training (no participant travel needed). Patient satisfaction was high in both interventions. For the SPT in particular, no negative effects on motivation and fatigue were observed and training difficulty and length were just right. Based on our findings, small adjustments in duration and frequency will make cognitive rehabilitation in advanced MS feasible, like it is in patients with less progressive disease courses.^10^

## Supplemental Material

sj-docx-1-mso-10.1177_20552173211064473 - Supplemental material for Feasibility of cognitive rehabilitation in patients with advanced multiple sclerosis: A pilot study Click here for additional data file. Supplemental material, sj-docx-1-mso-10.1177_20552173211064473 for Feasibility of cognitive rehabilitation in patients with advanced multiple sclerosis: A pilot study by Stefanos E Prouskas, Nancy D Chiaravalloti, Neeltje Kant, Karlene K Ball, Vincent de Groot, Bernard MJ Uitdehaag, Jeroen JG Geurts, Elizabeth A Kooij and Hanneke E Hulst in Multiple Sclerosis Journal – Experimental, Translational and Clinical
