# Supplementary material for: Feasibility of cognitive rehabilitation in patients with advanced multiple sclerosis: A pilot study
Source: Mult Scler J Exp Transl Clin. 2021 Dec 10;7(4):20552173211064473. doi: 10.1177/20552173211064473 (PMC8669124; doi:10.1177/20552173211064473)
Supplement: sj-docx-1-mso-10.1177_20552173211064473 - Supplemental material for Feasibility of cognitive rehabilitation in patients with advanced multiple sclerosis: A pilot study [file sj-docx-1-mso-10.1177_20552173211064473.docx]

# SUPPLEMENTARY MATERIAL

**Supplementary Method**

## Inclusion criteria

Inclusion criteria were: 1) clinically definite MS, according to McDonald (1) criteria (with PPMS or SPMS as an additional inclusion criterion), 2) 18-67 years of age (67 being the statutory retirement age in the Netherlands), 3) no history of (or current) drug abuse, neurological comorbidities, major psychiatric disorders such as bipolar disorder, psychosis, etc., 4) no changes in medication use (antidepressants and psychoactive drugs) in the last 4 weeks prior to participation, 5) sufficient communicative abilities and visual acuity to perform the neuropsychological testing, and the intervention. Disease severity was assessed by a validated EDSS-based questionnaire (2). Stratified randomization was used, with age, sex and educational level as stratification factors. Two strata per variable were used. Age was stratified as ≤ 60 years, or > 60 years, due to the older age of most residents of Nieuw Unicum. Education was stratified as a score < 6 on the Verhagen scale (i.e. a maximum education level of secondary education), or ≥ 6 on the Verhage scale (i.e. a maximum education level of collegiate or university tertiary education) (3).

**Interventions**

## Compensatory Memory Strategies Training

CST is a group intervention that is provided standard of care in patients in Nieuw Unicum. Patients that participated in CST in the last year as part of usual care were excluded from the current study. The program consists of psychoeducation and learning “tips and tricks” (i.e. compensation) to help patients cope with cognitive impairment in daily life (see table S1) (6). In addition to practical advice, emotional support is an important aspect of this group training. The sessions were given once a week for 90 minutes over a total period of 9 weeks, in two groups consisting of 4-5 patients per group.

Table S1**.** Compensation Strategies Training content per session.

**Compensation Strategies Training content per session**

1. Introductory session: ascertain expectations, set goals, get acquainted with group
2. The memory strategies: attention, time, repetition, association, organization, looking forward and back
3. External strategies: use external tools to remember things & Internal strategies: strategies to store information better in your long-term memory,
   or to learn something 'by heart'
4. Information about memory, memory problems, and the brain: what memory is,
   the origin of memory impairment in the brain
5. Repetition of internal strategies: attention, time management, association
6. Coping with memory problems: personal experiences
7. Coping styles: active tackling, avoidance, emotional expression
8. Application of taught material to personal situations: struggles and joys
9. Closing session, assessment, goal attainment: were expectations met?

## Speed of Processing Training

SPT is a computerized cognitive rehabilitation program that makes use of the UFOV® software (4), which focuses on IPS in such a way that increasingly more complex information can be processed within briefer periods of time. Similarly to training protocols from previous studies (5), patients trained twice a week, 60 minutes per session, for 5 weeks. SPT involved trainer-guided one-on-one practice of stimulus-based computer exercises. The 60 minutes per session included 45 minutes of training and a total of 15 minutes for filling out feasibility questionnaires, brief discussions about the training and the patient’s performance, and a brief pause halfway through the session. Due to motor impairment in the majority of patients included in this study, patients verbally indicated their responses to the researcher, who then clicked that response on the computer. Response time is unlimited and has therefore no effect on the training. For patients with RRMS, SPT is a self-explanatory training (5), while in the current study a researcher was present at every session to record the responses and ensure adherence to training instructions.

The training is tailored to the entry level of the individual patient (baseline level of IPS). Stimulus display duration, ranging from 50ms to 500ms, is the primary parameter during training. Decreasing stimulus display duration increases task demands and thus demands on IPS itself. Each training block consists of 16 trials. After two consecutive blocks with 75% success rate (i.e., ≥ 12 out of 16 trials correct per block) the task difficulty is increased by decreasing target display duration by 50ms. After two consecutive blocks with 75% success rate at shortest possible target display duration (50ms), the difficulty of the task is increased by 1) gradually increasing the complexity of the central task from present/absent, to identify, to same/different (Figure S1), and 2) adding a secondary task of localizing a peripheral stimulus, and further adding localization distractors in the peripheral field of view.


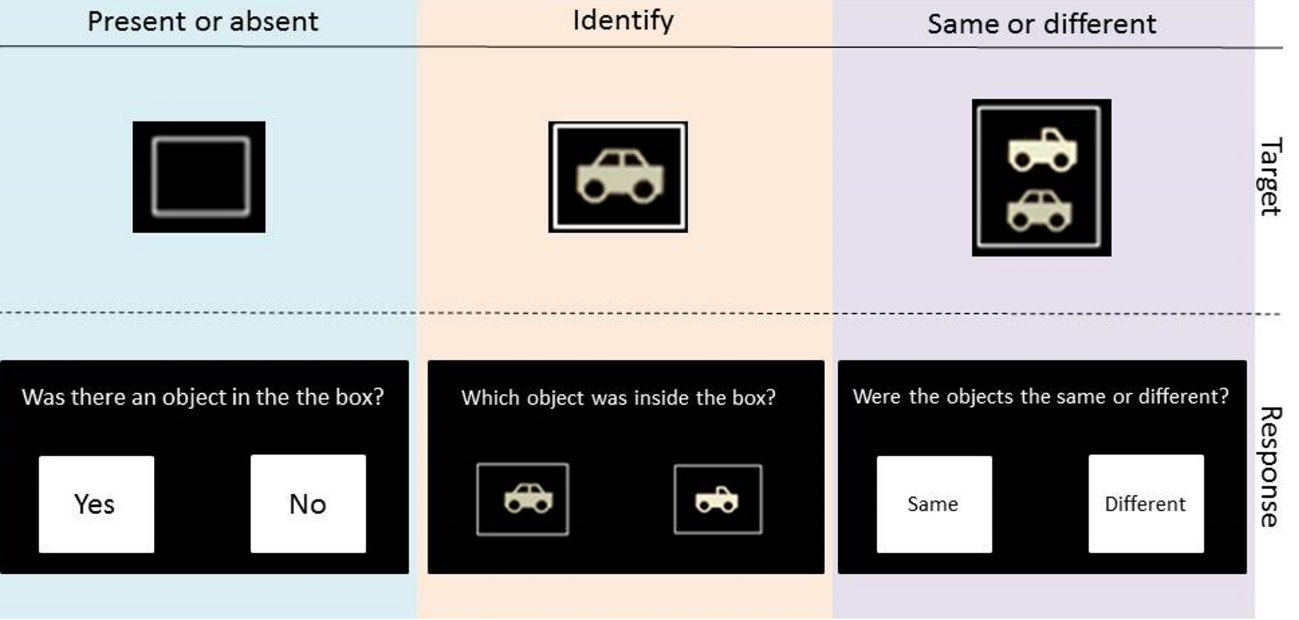


Fig. S1. Example of the three central tasks of the speed of processing training.

**
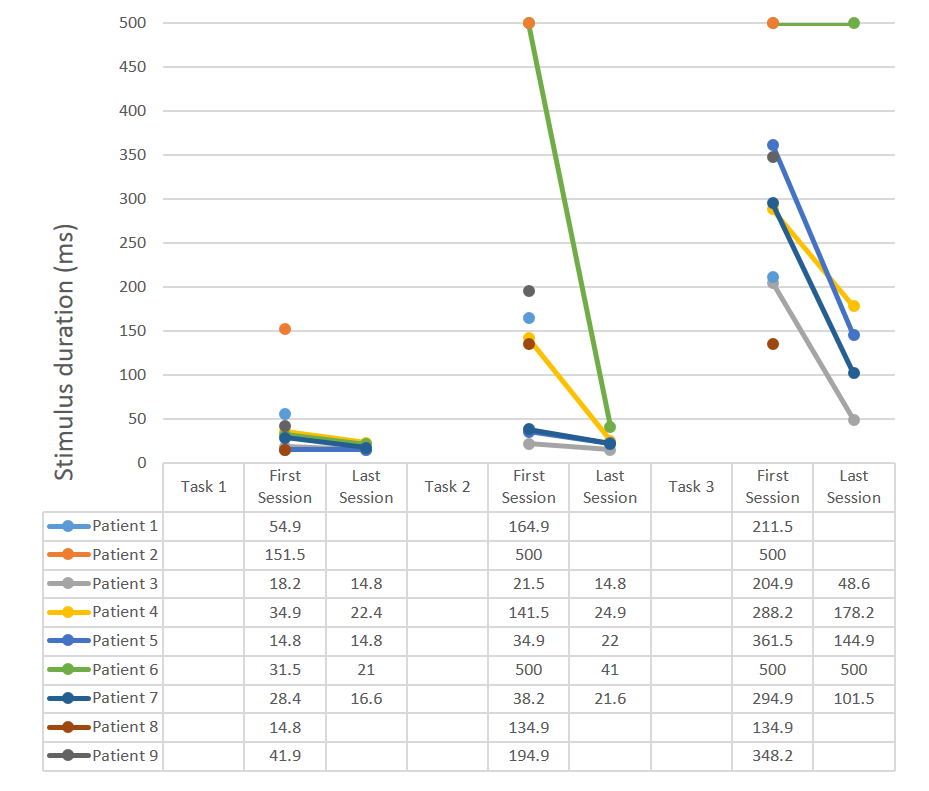
**

Fig. S2. SPT task performance assessment between first and last session. Four patients were unable to complete the assessment at the last session, due to extremely hot weather. Patients performance on the computer task was similar or better on the last session compared to the first (indicated by shorter stimulus duration). No participants scored worse after training.

## Neuropsychological measurement

Within one week before their first SPT or CST session (baseline) and within two weeks after their last session (follow-up), all patients underwent neuropsychological testing and completed questionnaires on depression, anxiety, fatigue, subjective cognitive functioning and QoL.

Neuropsychological testing consisted of a modified version of the minimal assessment of cognitive function in multiple sclerosis (MACFIMS) (7). The following tests were administered: CVLT-II, SDMT, WAIS-IV Symbol Search, WAIS-IV Cancellation, Stroop color word task, LLT, DKEFS sorting test, COWAT, WAIS-IV Digit Span, and JLO.

Additionally, the following questionnaires were administered: Hospital Anxiety and Depression Scale (HADS-NL Dutch version) (8), Checklist Individual Strength (CIS20-r) (9), the functional assessment of MS (FAMS) (10), and Cognitive Failures Questionnaire (CFQ) (11).

# Feasibility questionnaires

#### Rating of Speed of Processing Training *(self-report, single session)*

Participant number:…………. Session:………….. Date: ………………………………………..

*Mark on these lines how you are feeling right now.*

*Below the dotted line are two more questions for after the training session.*

How much energy do I have now?


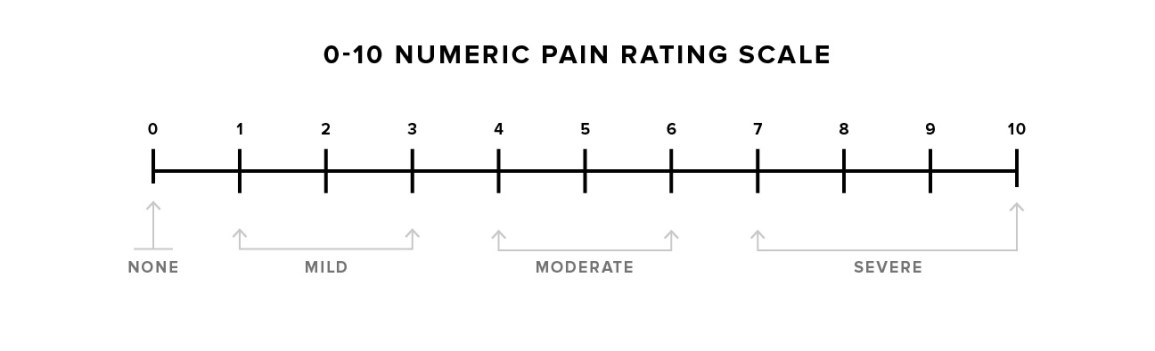


How motivated am I to start the training?


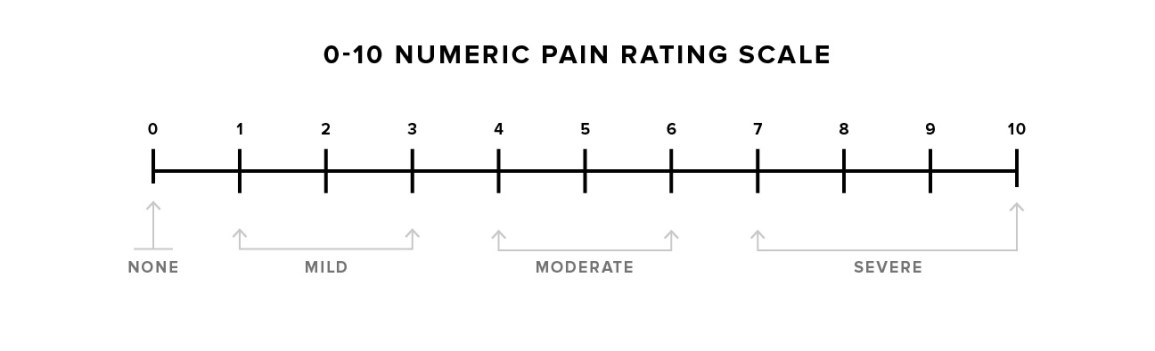


How much energy do I have right after training?


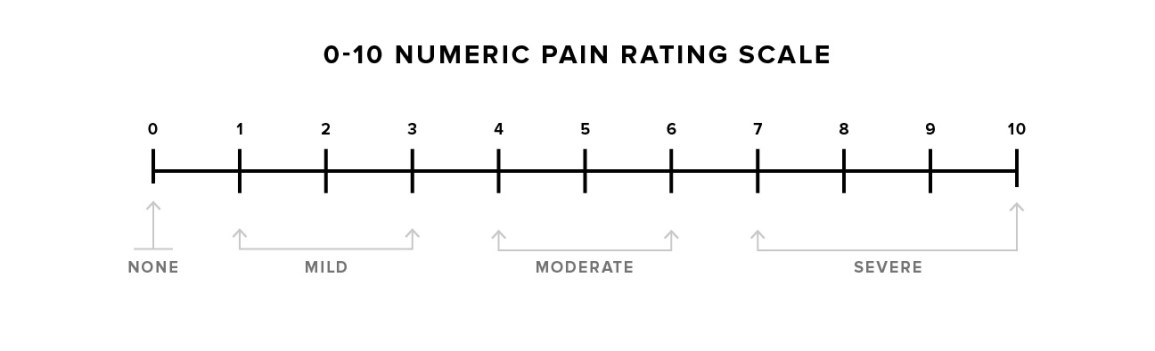


How do I think this training session went?


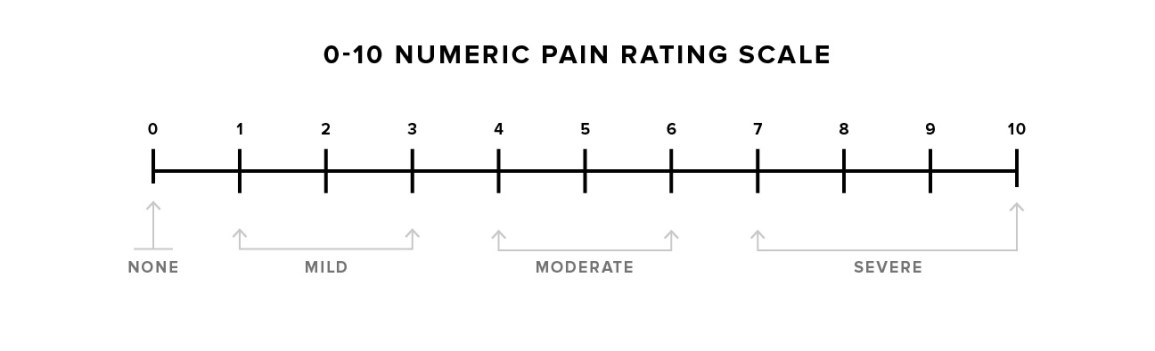


Comments:

..........................................................................................................................................

..........................................................................................................................................

#### Observations feasibility Speed of Processing Training *- (researcher, single session)*

Participant number:…………. Session:………….. Date: ………………………………………..

Present: yes / no (if not, reason:…………………………………………………………………………………..)

**Time on task: 1) ... min 2) ... min 3) ... min**

**Fill in after session:**

Fatigue:


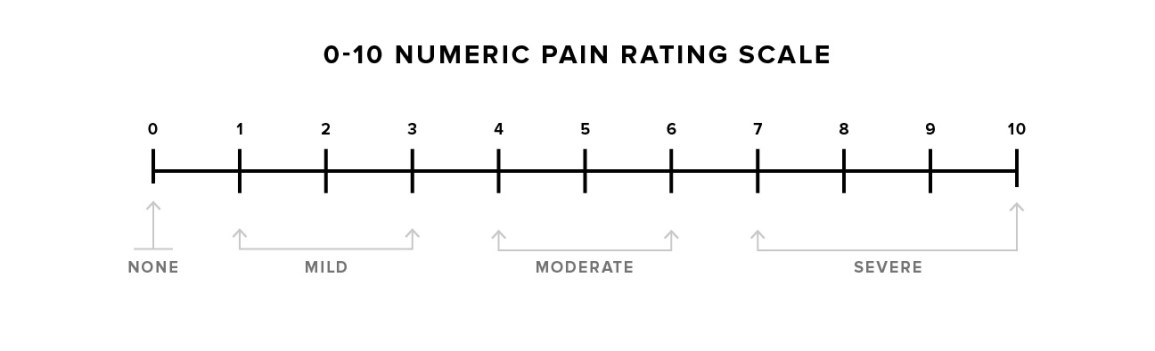
(e.g. yawning, closing eyes)

Comments:

......................................................................................................................................................................................................................................................................................................................................

Motivation:

(e.g. indicating that he/she doesn't feel like continuing, asking how long it will take)


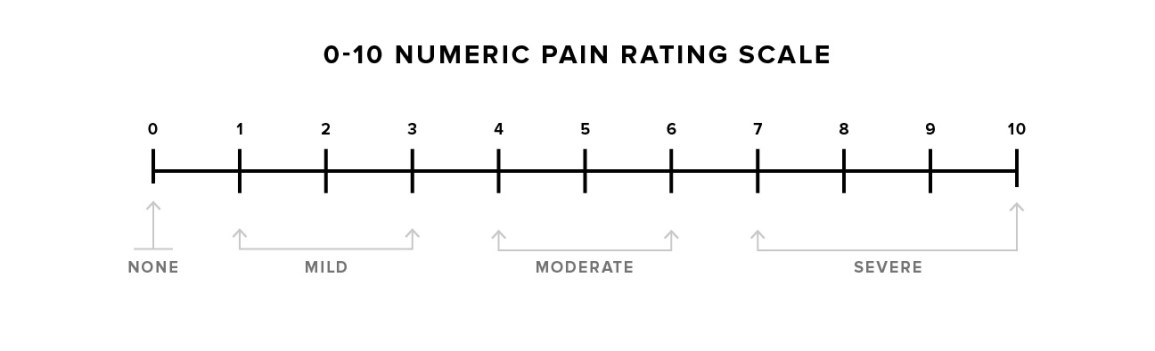


Comments:

......................................................................................................................................................................................................................................................................................................................................

Concentration:

(e.g. seems distracted, should be reminded to continue)


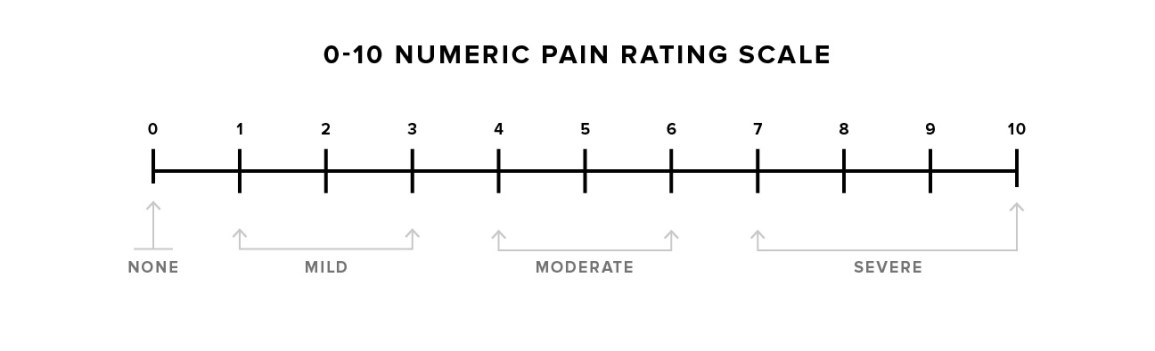


Comments:

......................................................................................................................................................................................................................................................................................................................................

General remarks:

......................................................................................................................................................................................................................................................................................................................................

#### General rating Speed of Processing Training *– patient-reported (before first session)*

Participant number:…………. Date: ………………………………………..

-What do you expect from the training?

.........................................................................................................................................................................................................................................................................................................................................................................................................................................................................................................

*Tick on the line how you rate this training*

-How motivated are you for this training?


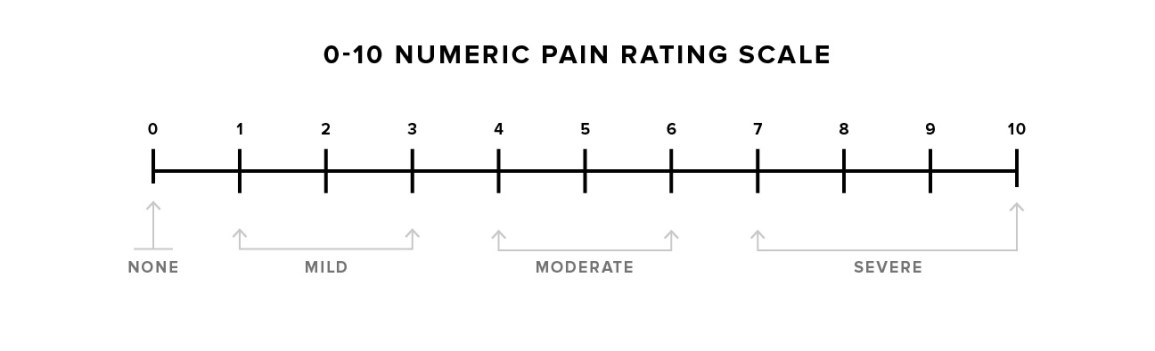


*totally unmotivated very motivated*

#### General rating Speed of Processing Training *– researcher (after first session)*

Researcher:………………………………………… Date:…………………………………

Participant number: …………………………

-General remarks / details / highlights:

......................................................................................................................................................................................................................................................................................................................................


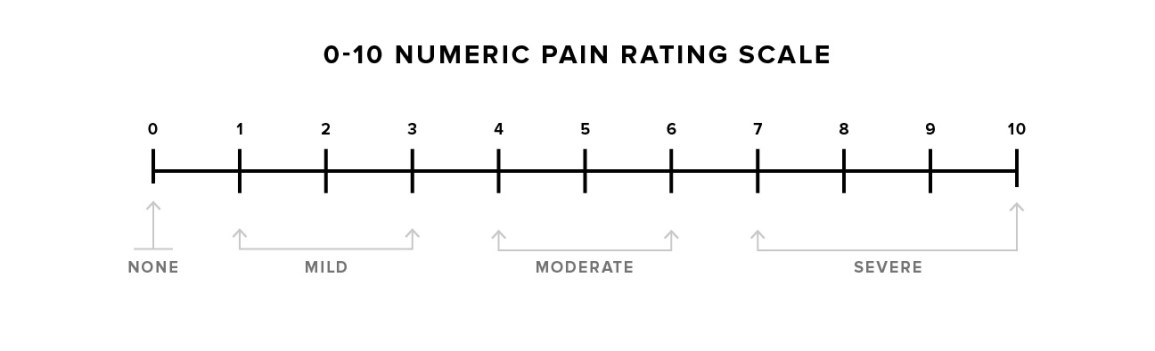


-Level of training:

*Too easy just right too difficult*

Comments:

......................................................................................................................................................................................................................................................................................................................................

-Participant burden:


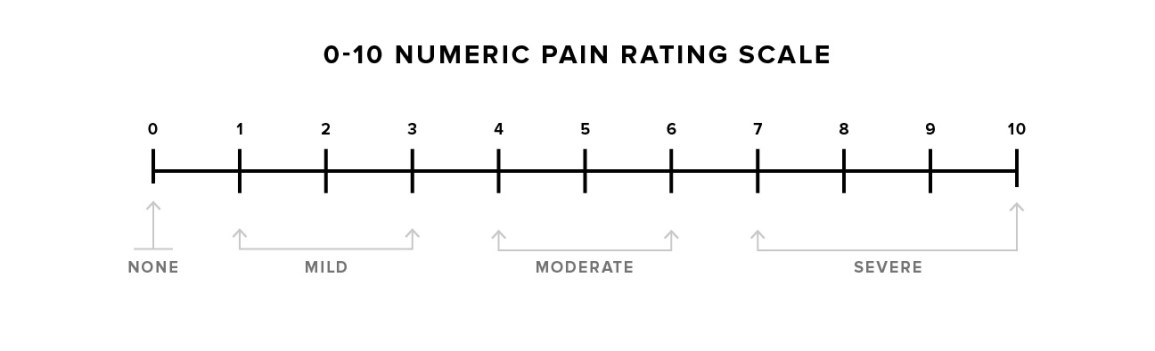
(is it tiresome, confronting)

*not burdensome at all very burdensome*

Comments: ......................................................................................................................................................................................................................................................................................................................................

-Motivation:


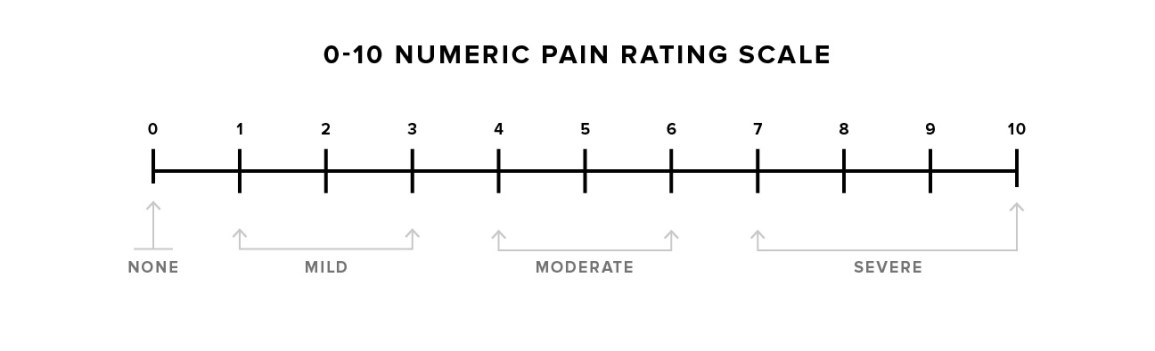


*totally unmotivated very motivated*

Comments:

......................................................................................................................................................................................................................................................................................................................................

-Feasibility


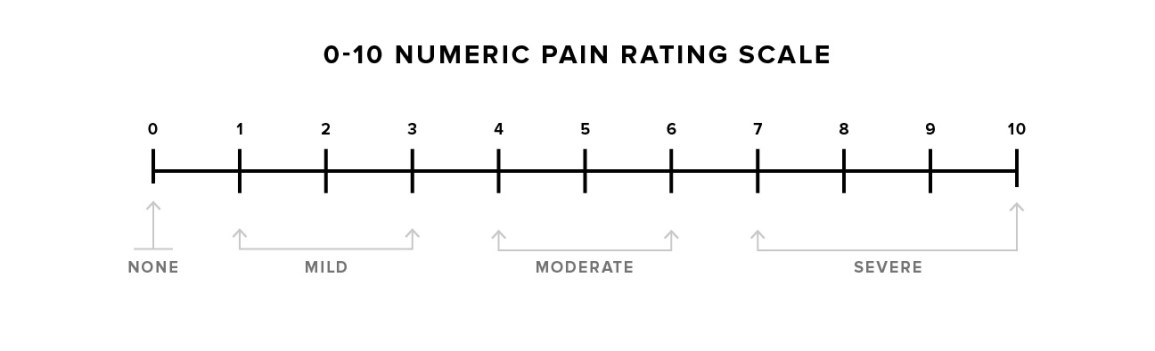
(understanding instructions, intelligibility, handling material)

*totally unfeasible easily feasible*

Comments:

................................................................................................................................................................... ...................................................................................................................................................................

#### General rating Speed of Processing Training *– patient-reported (after last session)*

Participant number:…………. Date: ………………………………………..

-How did you experience the training?

..........................................................................................................................................................................................................................................................................................................................................

*Tick on the lines how you rate this training*

-How tiring did you find the training?


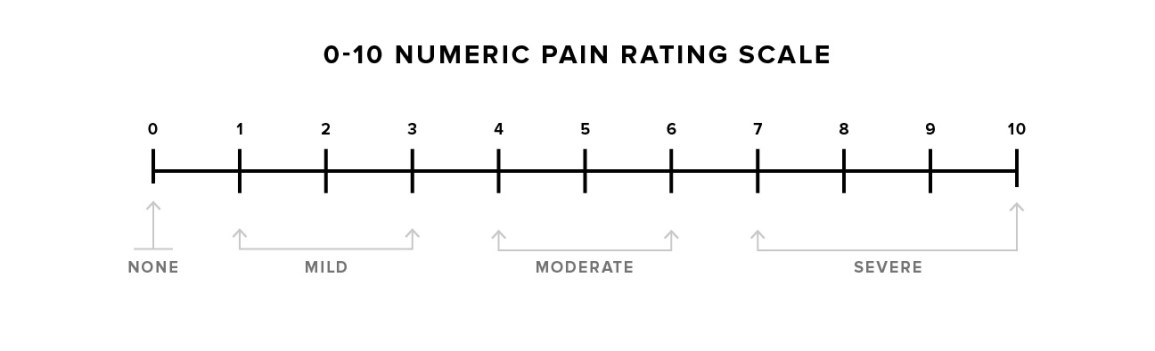


*not tiring at all very exhausting*

-How well were you able to maintain your concentration during training?


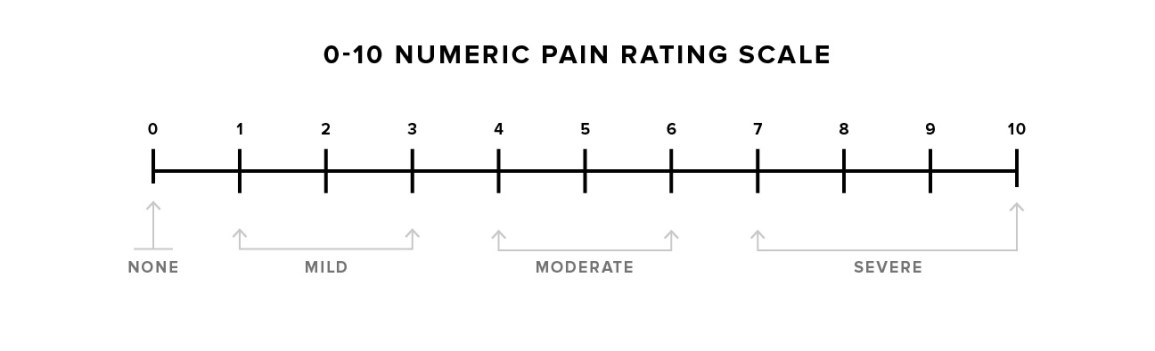


*not at all perfectly*

-How clear were the instructions?


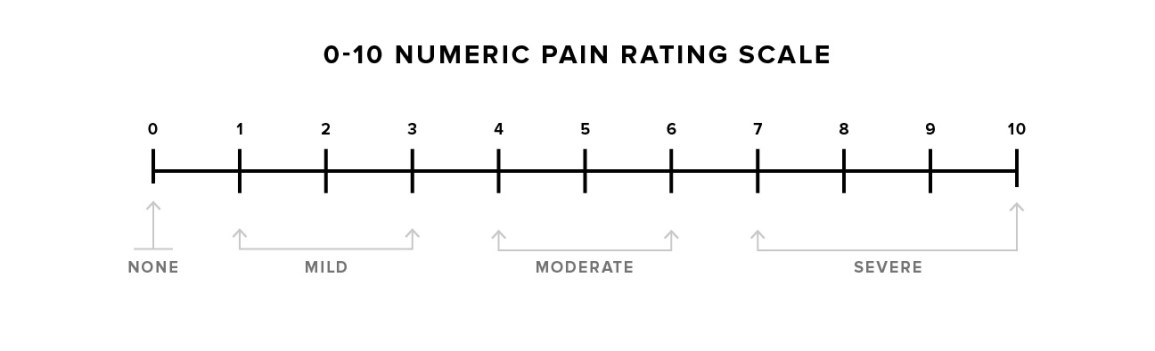


*Not clear at all fully clear*

-How difficult did you find the exercises?


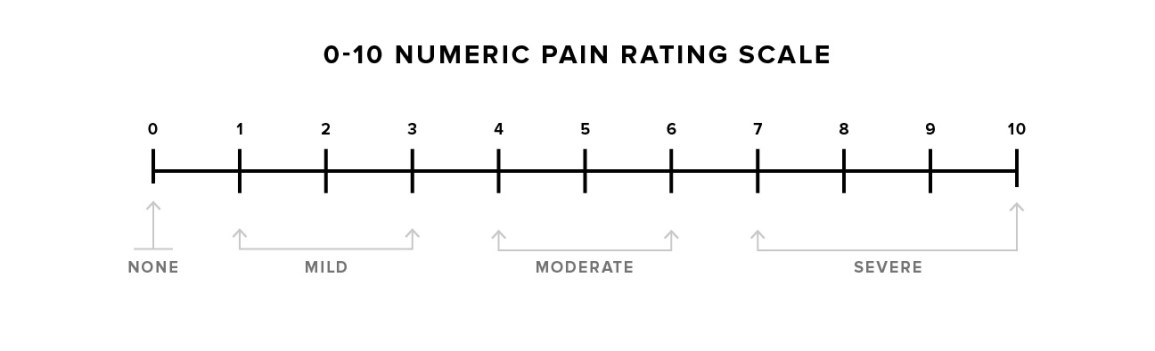


*Very easy very difficult*

-How accessible was the material to you?

(e.g. computer use, mouse operation, readability/visibility)


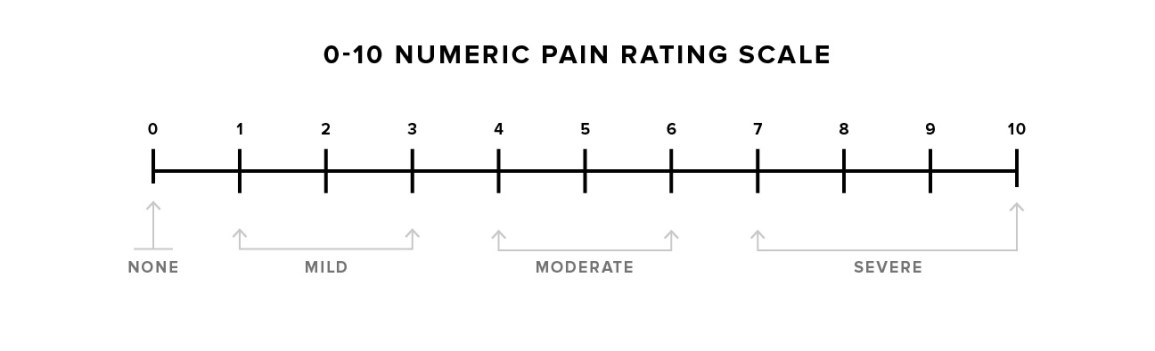


*Not accessible at all perfectly accessible*

-What did you think of the duration of the training?


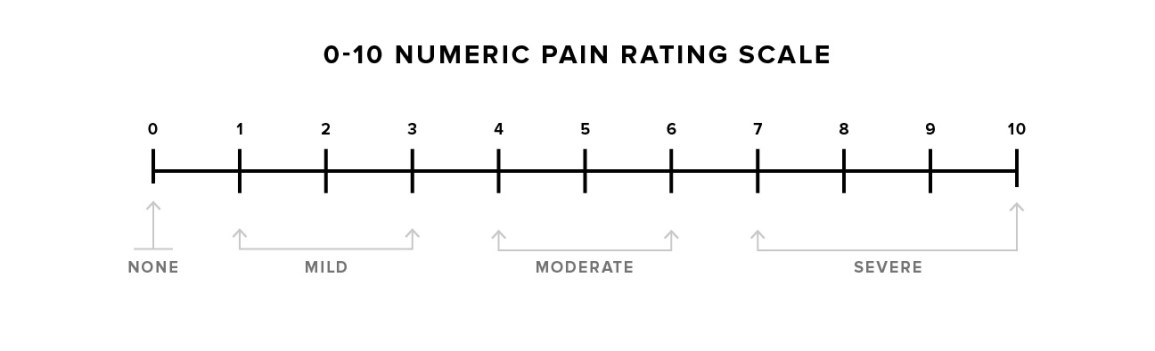


*Too short just right too long*

#### General rating Speed of Processing Training *– researcher (after last session)*

Researcher:………………………………………… Date:…………………………………

Participant number: …………………………

-What did you think of the training for this participant?

.........................................................................................................................................................................................................................................................................................................................................................................................................................................................................................................

...................................................................................................................................................................

-What did you think of the duration of the training for the participant?


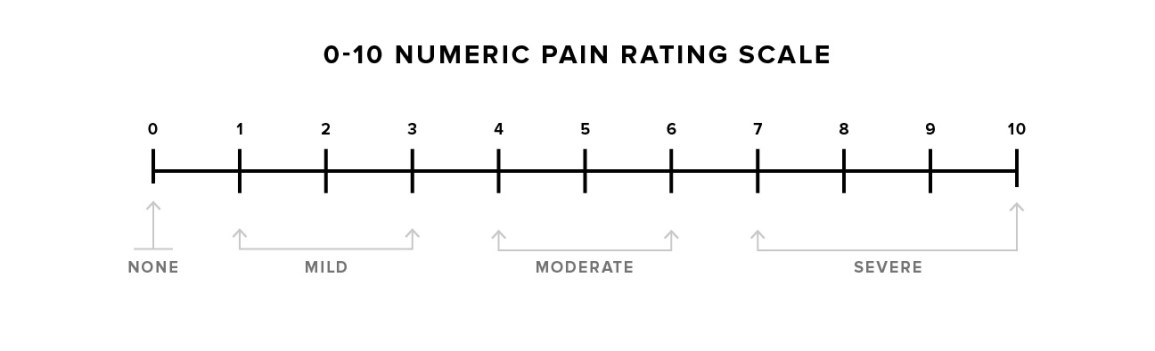


*Too short just right too long*

-How tiring was the training for the participant?


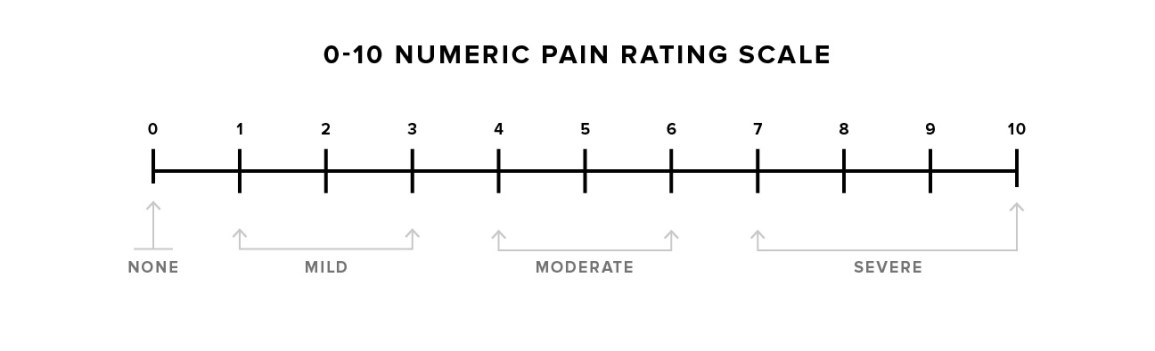


*not tiring at all very exhausting*

-How well was the participant able to maintain concentration during the training?


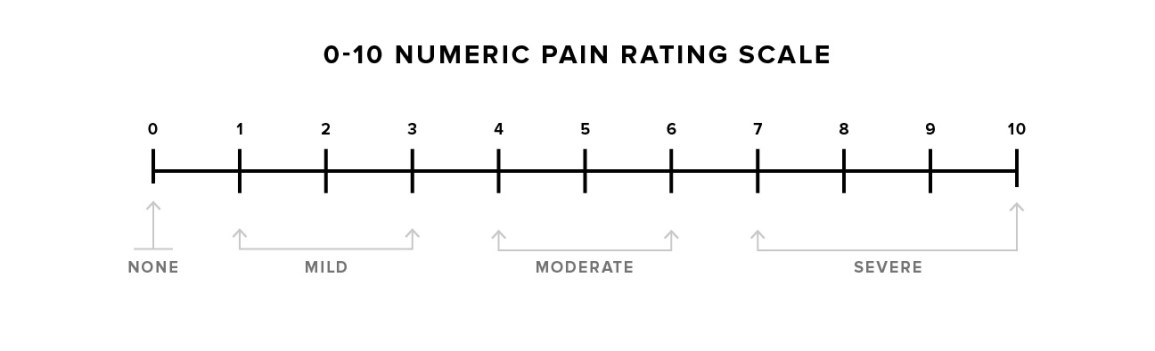


*not at all perfectly*

- How clear were the instructions?


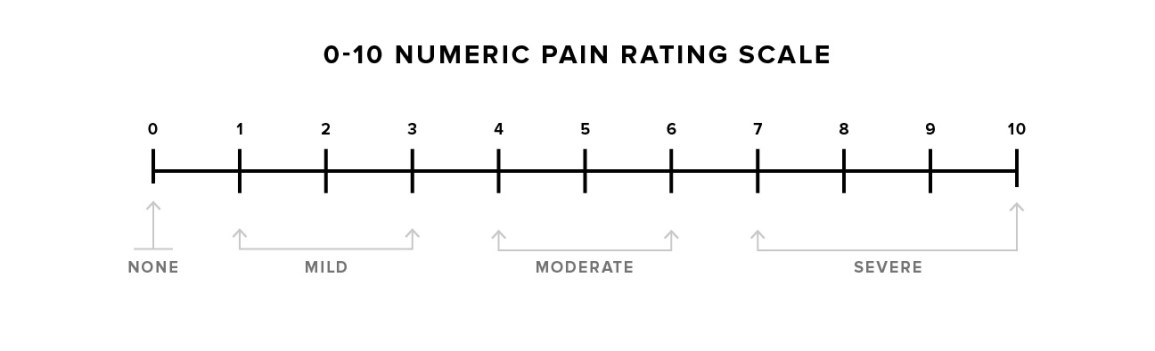


*Not clear at all fully clear*

- How difficult were the exercises for the participant?


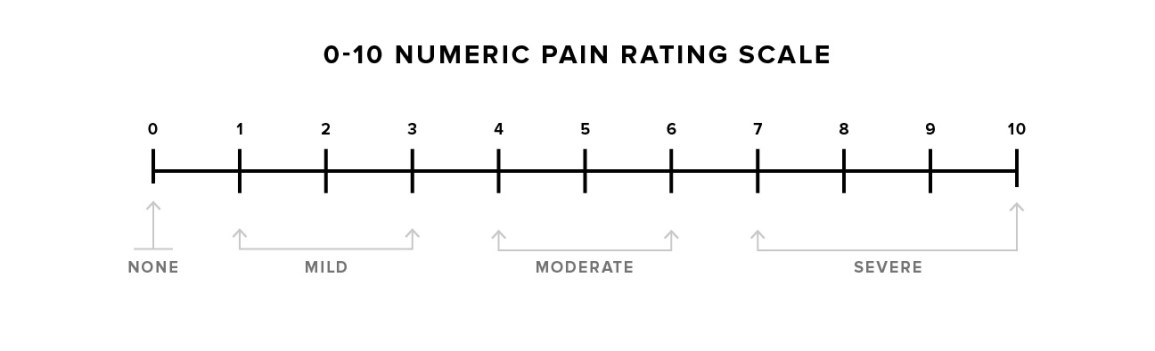


*Very easy very difficult*

-How accessible was the material to the participant?

(e.g. computer use, mouse operation, readability/visibility)


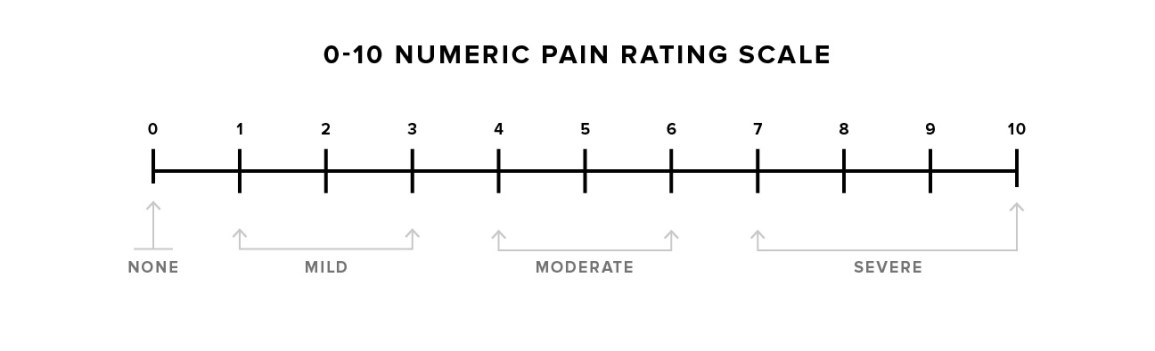


*Not accessible at all perfectly accessible*

## References

1. Thompson AJ, Banwell BL, Barkhof F, Carroll WM, Coetzee T, Comi G, et al. Diagnosis of multiple sclerosis: 2017 revisions of the McDonald criteria. The Lancet Neurology. 2018;17(2):162-73.

2. Lechner-Scott J, Kappos L, Hofman M, Polman CH, Ronner H, Montalban X, et al. Can the Expanded Disability Status Scale be assessed by telephone? Multiple Sclerosis Journal. 2003;9(2):154-9.

3. Verhage F. Intelligentie en leeftijd bij volwassenen en bejaarden. Groningen1964.

4. Ball K, Owsley C. The Useful Field of View Test: A new technique for evaluating age-related declines in visual function1993. 71-9 p.

5. Chiaravalloti ND, Goverover Y, Costa SL, DeLuca J. A Pilot Study Examining Speed of Processing Training (SPT) to Improve Processing Speed in Persons With Multiple Sclerosis. Frontiers in Neurology. 2018;9(685).

6. van Kessel M, Fasotti L, Berg I, van Hout M, Wekking E. Training geheugenstrategieën. Amsterdam: Uitgeverij Boom; 2010. 177 p.

7. Benedict RHB, Cookfair D, Gavett R, Gunther M, Munschauer F, Garg N, et al. Validity of the minimal assessment of cognitive function in multiple sclerosis (MACFIMS). Journal of the International Neuropsychological Society. 2006;12(04).

8. Zigmond AS, Snaith RP. The Hospital Anxiety and Depression Scale. Acta Psychiatrica Scandinavica. 1983;67(6):361-70.

9. Vercoulen JHMM, Swanink CMA, Fennis JFM, Galama JMD, van der Meer JWM, Bleijenberg G. Dimensional assessment of chronic fatigue syndrome. Journal of Psychosomatic Research. 1994;38(5):383-92.

10. Cella DF, Dineen K, Arnason B, Reder A, Webster KA, Karabatsos G, et al. Validation of the Functional Assessment of Multiple Sclerosis quality of life instrument. Neurology. 1996;47(1):129.

11. Broadbent DE, Cooper PF, FitzGerald P, Parkes KR. The Cognitive Failures Questionnaire (CFQ) and its correlates. British Journal of Clinical Psychology. 1982;21(1):1-16.
